# The *Arabidopsis* epigenetic regulator ICU11 as an accessory protein of Polycomb Repressive Complex 2

**DOI:** 10.1073/pnas.1920621117

**Published:** 2020-06-29

**Authors:** Rebecca H. Bloomer, Claire E. Hutchison, Isabel Bäurle, James Walker, Xiaofeng Fang, Pumi Perera, Christos N. Velanis, Serin Gümüs, Christos Spanos, Juri Rappsilber, Xiaoqi Feng, Justin Goodrich, Caroline Dean

**Affiliations:** ^a^Department of Cell and Developmental Biology, John Innes Centre, Colney Lane, NR47UH Norwich, United Kingdom;; ^b^Institute of Molecular Plant Sciences, University of Edinburgh, Max Born Crescent, EH9 3BF Edinburgh, United Kingdom;; ^c^Faculty of Biotechnology, Hochschule Mannheim, 68163 Mannheim, Germany;; ^d^Wellcome Centre for Cell Biology, University of Edinburgh, Max Born Crescent, EH9 3BF Edinburgh, United Kingdom;; ^e^Institute of Biotechnology, Technische Universität Berlin, 13355 Berlin, Germany

**Keywords:** epigenetic, ICU11, Polycomb, chromatin

## Abstract

Molecular mechanisms enabling the switching and maintenance of epigenetic states are not fully understood. Distinct histone modifications are often associated with ON/OFF epigenetic states, but how these states are stably maintained through DNA replication, yet in certain situations switch from one to another remains unclear. Here, we address this problem through identification of *Arabidopsis* INCURVATA11 (ICU11) as a Polycomb Repressive Complex 2 accessory protein. ICU11 robustly immunoprecipitated in vivo with PRC2 core components and the accessory proteins, EMBRYONIC FLOWER 1 (EMF1), LIKE HETEROCHROMATIN PROTEIN1 (LHP1), and TELOMERE_REPEAT_BINDING FACTORS (TRBs). *ICU11* encodes a 2-oxoglutarate–dependent dioxygenase, an activity associated with histone demethylation in other organisms, and mutant plants show defects in multiple aspects of the *Arabidopsis* epigenome. To investigate its primary molecular function we identified the *Arabidopsis FLOWERING LOCUS C* (*FLC*) as a direct target and found *icu11* disrupted the cold-induced, Polycomb-mediated silencing underlying vernalization. *icu11* prevented reduction in H3K36me3 levels normally seen during the early cold phase, supporting a role for ICU11 in H3K36me3 demethylation. This was coincident with an attenuation of H3K27me3 at the internal nucleation site in *FLC*, and reduction in H3K27me3 levels across the body of the gene after plants were returned to the warm. Thus, ICU11 is required for the cold-induced epigenetic switching between the mutually exclusive chromatin states at *FLC*, from the active H3K36me3 state to the silenced H3K27me3 state. These data support the importance of physical coupling of histone modification activities to promote epigenetic switching between opposing chromatin states.

## Significance

Epigenetic regulation of gene expression is associated with switching between chromatin states characterized by distinct histone modifications. Polycomb/Trithorax regulation involves the mutually exclusive H3K27me3/H3K36me3 modifications, but how these states are faithfully inherited through DNA replication, yet can switch from one to another, is still poorly understood. One mechanism that would aid switching is the association of histone methyltransferases with factors demethylating the opposing histone modification. Here, we show that an *Arabidopsis* 2-oxoglutarate–dependent dioxygenase, an activity associated with histone demethylation in other organisms, physically associates with the Polycomb Repressive Complex 2. We propose that physical association of histone methylation/demethylation activities will be generally important to coordinate switching between chromatin states.

Epigenetic silencing is mediated by conserved histone-based mechanisms in many organisms. Polycomb Repressive Complex 2 (PRC2) delivers a H3K27me3-based silencing that plays major roles in developmental and environmental epigenetic regulation (1–3). The PRC2 core components are conserved between organisms and associate with more diverse PRC2 accessory proteins that recognize sequence-specific or chromatin features such as CpG islands (4–13). A well-studied PRC2 target in *Arabidopsis* is the floral repressor locus, *FLOWERING LOCUS C* (*FLC*) (14). The prolonged cold of winter epigenetically silences the locus enabling expression in the spring of the genes required for the meristem to adopt a floral fate (15–18). *FLC* silencing occurs in a two-step cis-mediated PRC2 switching mechanism mediated by distinct complexes. During the cold, a PRC2 containing SWINGER (SWN) and associated with the accessory proteins VERNALIZATION5 (VRN5) and VERNALIZATION INSENSITIVE3 (VIN3) nucleates H3K27me3 silencing at a small intragenic site and this confers a metastable silencing (19). After return to warm, a PRC2 containing CURLY LEAF (CLF) and LHP1 mediate spreading of H3K27me3 silencing across the gene body, and this confers long-term stable silencing (19). Resetting *FLC* expression then occurs as the embryos develop to ensure vernalization is required each generation, a process requiring the H3K27me3 demethylase, EARLY FLOWERING 6 (ELF6) (20).

Modeling of the mechanism at *FLC* has provided a generic view of how opposing chromatin states, marked by a transcriptionally active H3K36me3 state and a silenced H3K27me3 state, can provide epigenetic stability, yet switch from one to another (1, 15, 21). This mechanism involves positive feedbacks to maintain each state and nonlinearity in the interaction mechanisms (22). Physical coupling of methylase and demethylase activities has been proposed to contribute to cooperativity in the system, a prediction partly validated for the *Arabidopsis* system by the finding that the H3K27me3 demethylase ELF6 physically interacted in vivo with the H3K36me3 methyltransferase SET DOMAIN GROUP 8 (SDG8) (23). Whether there was a similar physical interaction linking H3K36me3 demethylation with the Polycomb methyltransferases was not known.

Here, through analysis of a *Ds* transposon-tagged *Arabidopsis* mutation showing pleiotropic developmental phenotypes we identify an activity associated with H3K36me3 demethylation. The mutation was initially called *wavy leaves and cotyledons furled back* (*wlc-1*) and was identified as an early flowering, deformed leaf mutant in the Landsberg *erecta* genotype (24). Cloning revealed the gene (At1g22950) corresponds to the recently described *ICU11* locus (25), so the mutant is hereafter referred to as *icu11-3*. ICU11 is part of a small family of genes in the *Arabidopsis* genome with partially redundant functions. *icu11* mutants misexpress many developmental regulators, share many phenotypes with *embryonic flower* mutants, and genetic analysis linked ICU11 activity with PRC2 function (25). Here, we extend this understanding and show that ICU11 robustly associates with PRC2 components in plants, and when defective, PRC2-mediated repression is compromised. Through analysis of a direct target, the floral repressor locus *FLC*, we find that ICU11 facilitates H3K36me3 demethylation and promotes the switch to the Polycomb H3K27me3 silenced state. *icu11* also shows other subtle epigenetic changes, so we propose that perturbed histone demethylation causes increased transcriptional activity of ICU11 targets with direct effects on PRC2 silencing and indirect effects on the wider *Arabidopsis* epigenome.

## Results

### *Ds* Insertion into At1g22950 Results in Weak PcG Mutant Phenotypes.

**Fig. 1.**
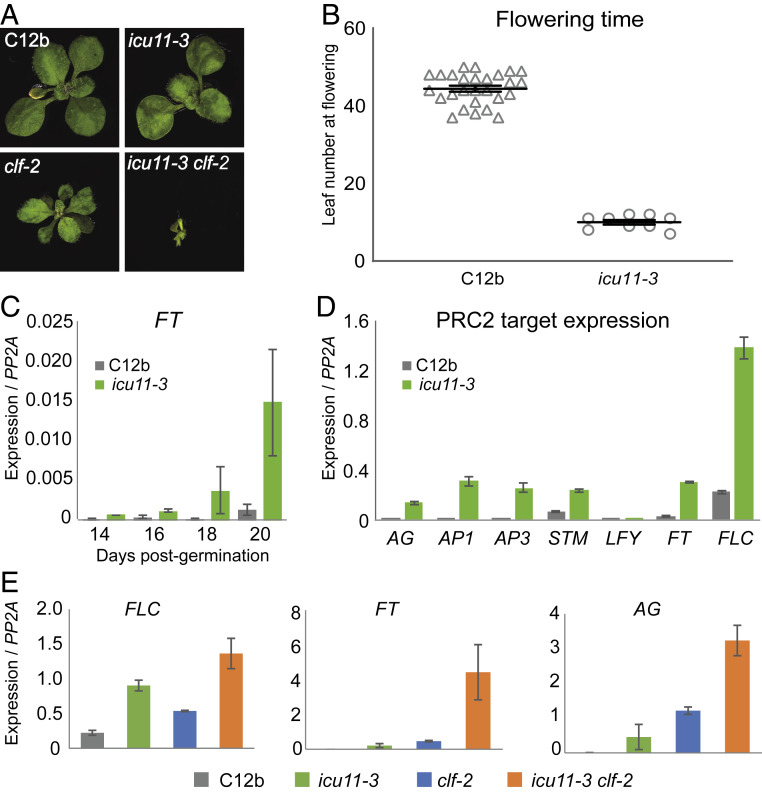
*icu11-3* phenotypes are reminiscent of mutants defective in PRC2. (*A*) Morphological phenotypes of WT line C12b, the *icu11*-3 and *clf-2* mutants, and synergistic interaction in *icu11-3 clf-2* double mutant. (*B*) Flowering is significantly accelerated under short-day conditions in *icu11-3* (*P* < 0.0001, unpaired Student’s *t* test). Error bars represent SEM (*n* = 9 [*icu11-3*]; *n* = 27 [WT]). (*C*–*E*) Mean gene expression in C12b WT, *icu11-3*, *clf-2*, and *icu11-3 clf-2* double-mutant lines. Target gene expression is normalized to the housekeeping gene *PP2A*; error bars represent SEM for three biological replicates. (*C*) The floral activator *FT* is overexpressed in early seedling development in *icu11-3* compared with WT under short-day conditions. (*D*) The *icu11-3* mutant overexpresses PRC2 target genes during early seedling development. (*E*) The *icu11-3 clf-2* double mutant synergistically overexpresses PRC2 targets compared with either single mutant.

The *wlc-1* mutant, hereafter *icu11-3*, was identified in an *Ac*/*D*s transposon-tagging mutagenesis screen (24) through its phenotypes, which include small size, pronounced cotyledon and leaf curling and early flowering in short days (Fig. 1 *A* and *B*). Subsequent cloning revealed it to be ICU11, a 397-amino acid protein of unknown function (At1G22950, TAIR10), with the InterPro EMBL-EBI protein domain database predicting an Fe^2+^/2-oxoglutarate–dependent dioxygenase (2OG) domain with homology to *Arabidopsis* prolyl-4-hydroxylases (P4Hs) and alpha ketoglutarate-dependent dioxygenase Bs (AlkBs) (26). In *icu11-3*, the insertion of the transposed *Ds* into the fifth exon introduces a premature stop codon, truncating ICU11 before the predicted enzymatic domain (*SI Appendix*, Fig. S1*A*). In addition, the *icu11-3* mutant expresses approximately fourfold less *ICU11* at the mRNA level (*SI Appendix*, Fig. S1*B*). Transformation of the *icu11-3* mutant with genomic *ICU11* alone, or genomic *ICU11* with C-terminal enhanced green fluorescent protein (eGFP) or 3xHA fusion constructs complemented *icu11-3*, rescuing the observed morphological defects (*SI Appendix*, Figs. S1*C* and S2 *A* and *B*) and the misexpression of target genes in young seedlings (*SI Appendix*, Figs. S1*D* and S2 *C and D*). In addition, revertants were generated through *Ac*-induced remobilization of *Ds* to generate wild-type (WT) plants and fully stable mutant plants; in the latter the excision had led to a frame shift in the coding sequence. *ICU11* is widely expressed, appearing elevated during later plant development (*SI Appendix*, Fig. S1*E*), while pICU11::ICU11-eGFP constructs showed broad expression of the protein in roots, nuclear localization at the subcellular level and association with metaphase chromosomes (*SI Appendix*, Fig. S1*F*).

To investigate the early flowering of *icu11-3* in short days, we compared the expression of the floral activator *FT*, a known PRC2 target, during early seedling development under short-day growth conditions. *FT* was up-regulated in *icu11-3* compared with the progenitor line (C12b), with the overexpression phenotype becoming more pronounced with seedling age (Fig. 1*C*). To investigate the potential misregulation of PRC2 targets more generally, we performed quantitative RT-PCR (qRT-PCR) on RNA extracted from 14-d-old seedlings of both *icu11-3* and the progenitor line (Fig. 1*D*) and found the *icu11-3* mutant overexpresses a range of PRC2 target genes compared with WT, including the reproductive transition regulators *FT* and *FLC*, floral genes *AG*, *AP1*, and *AP3*, and the shoot meristem maintenance gene *STM*. We crossed *icu11-3* with *clf-2* and compared morphological and gene expression phenotypes in the single and double mutants. As found previously, double mutants exhibit severe morphological defects (Fig. 1*A*) and higher up-regulation of the flowering targets *FLC, FT*, and *AG* compared with either single mutant alone (Fig. 1*E*), similar to combination of *clf* with mutations in other PRC2 accessory proteins such as *lhp1* (27).

### ICU11 Is a PRC2 Accessory Protein.

To investigate ICU11 function further, we performed coimmunoprecipitation/mass spectrometry (coIP-MS) experiments using 3xHA- and GFP-tagged ICU11 proteins as bait. Our IP-MS revealed core PRC2 complex components CLF, SWN, FERTILIZATION INDEPENDENT ENDOSPERM (FIE), MULTICOPY SUPPRESSOR OF IRA1 (MSI), and EMBRYONIC FLOWER (EMF)2 and accessory proteins EMF1, LHP1, and TELOMERE-REPEAT-BINDING (TRB) 1–3, as ICU11 interactors. EMF1 and LHP1 have been found in several studies as direct interactors of PRC2 (28, 29), and TRBs have been found associated with CLF and SWN (11). JMJ14, a H3K4me3 demethylase (30), was also detected in the ICU11-enriched peptides. Reciprocal coIP-MS with CLF-GFP, SWN-GFP, and EMF1-FLAG confirmed the interaction between ICU11 and PRC2 (Table 1 and Dataset S1). Interestingly, we did not find ICU11 interacting with VIN3 or its homologs VRN5 and VEL1, reinforcing the view of distinct Polycomb complexes operating over different spatial and temporal timescales.

**Table 1.** ICU11 associates with the PRC2

	Protein	*35S::GFP*	*gICU11-GFP icu11-3*	*icu11-3*	*gICU11-HA icu11-3*	Col-0	*EMF1-3XFLAG emf1*	*35S::GFP*	*35S:GFP-CLF clf-50*	*gSWN-GFP*
Core PRC2	ICU11	0–0	13–19	0–0	12–11	0–0	11–20	0–0	13–2	5–5
	EMF2	0–0	11–4	0–0	29–0	0–0	2–22	0–0	33–18	4–36
	MSI1	0–0	12–6	0–0	25–2	3–2	6–18	2–0	22–18	6–26
	FIE	0–0	10–1	0–0	18–0	0–0	5–17	2–0	23–22	6–25
	SWN	0–0	10–0	0–0	27–0	0–0	0–26	0–0	11–0	21–68
	CLF	0–0	7–0	0–0	26–0	0–0	3–17	9–0	75–46	8–4
Accessory	EMF1	0–0	5–3	0–0	26–2	0–0	6–40	0–0	46–17	2–37
	LHP1	0–0	2–1	0–0	11–0	0–0	2–14	0–0	22–8	3–18
	TRB1	0–0	5–9	0–0	12–2	0–0	0–1	0–0	0–0	0–0
	TRB2	0–0	9–5	0–0	15–1	0–0	1–11	0–0	11–3	2–10
	TRB3	0–0	9–6	0–0	15–3	0–0	3–12	0–0	9–4	2–7
	VRN5	0–0	0–0	0–0	0–0	0–0	0–0	0–0	24–13	0–24
	VEL1	0–0	0–0	0–0	0–0	0–0	0–0	0–0	41–28	0–46
	ALL PEPTIDES	787–1817	1286–7239	2944–3524	5481–6267	11483–16737	11170–13095	6705–2489	7376–7714	5127–6855

The numbers indicate uniquely identified peptides from each protein found by mass spectrometry in two independent experiments. The total number of peptides identified in each experiment is also indicated at the bottom of the columns. PRC2 core components are shown at the top, with accessory components below. Columns are paired with control on left and immunoprecipitated to the right. For example, 35S:GFP samples in column 1 were the control for the ICU11-GFP samples in column2 in the gICU11-GFP analysis. 35S:GFP-CLF *clf50* and gSWN-GFP share the 35S::GFP control. The full list of proteins identified is presented as an excel sheet in Dataset S1.

### ICU11 as a Putative Histone Demethylase.

**Fig. 2.**
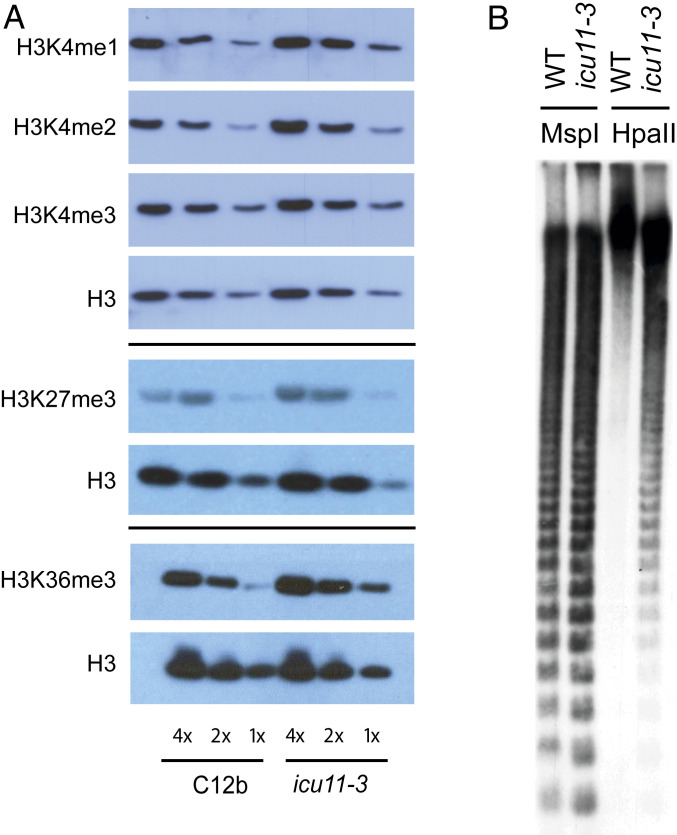
*icu11-3* affects global histone methylation and DNA methylation at the pericentromeric cen180 repeats. (*A*) The *icu11-3* mutant shows elevation of H3K36me3 and H3K4 methylation, but no change in H3K27me3 compared with WT C12b. Histones were prepared from a nuclear extract from 14-d-old seedlings and serially diluted; Western blots are shown with total histone H3 from the same extraction as loading controls. (*B*) Southern blot analysis of methylation at the pericentromeric cen180 repeat region. Genomic DNA digested with the methylation-insensitive restriction enzyme MspI (control) and methylation-sensitive enzyme HpaII indicates slightly reduced DNA methylation over centromeric repeats in *icu11-3*.

To further investigate the role of ICU11 in epigenetic regulation we used Western blots to determine levels of histone modifications associated with active chromatin (H3K4me1, H3K4me2, H3K4me3; H3K36me3) and those associated with silenced chromatin (H3K27me3) in WT and *icu11-3* 14-d-old seedlings. We observed a small increase in H3K4me2/me3, and H3K36me3 in *icu11-3* relative to WT (Fig. 2*A* and *SI Appendix*, Figs. S3 and S4). These data, together with the analysis of *FLC* derepression in the *icu11-3* mutant, raised the possibility that a primary activity of ICU11 was demethylation of histone modifications associated with active chromatin states, for example, H3K4me2/3 and H3K36me3. Loss of one protein may destabilize the whole-protein complex that contains putative demethylases specific for different histone modifications. A combination of activities, e.g., ICU11 and JMJ14 in the whole complex would then link demethylation of active histone modifications at multiple sites on the histone tail. This linking of activities in vivo means in vitro analyses are the best way to define the specific activity of different demethylases. The 2OGD domain of ICU11 falls within the same enzymatic superfamily as Jumonji C-domain histone demethylases. Despite considerable effort using in vitro assays using recombinant protein and commercial histones, or by colocalization assays of demethylation and expressed ICU11 protein in *Nicotiana benthamiana* transient assays, we could not generate reproducible data to clearly support a direct H3K4 or K36me3 demethylase role for ICU11. This is not uncommon for this family of proteins and may indicate that ICU11 requires accessory proteins or specific posttranslational modification for histone demethylase activity. It is also possible that ICU11 modifies another component of the complex, which then allosterically influences histone demethylation.

### DNA Methylation Is Subtly Changed in *icu11-3*.

The wide range of phenotypes in *icu11* mutants prompted us to look for changes in other epigenetic marks. In plants, DNA methylation occurs in CG, CHG, and CHH contexts (H = A, T, or C). CG methylation is broadly distributed, present in both gene bodies and repeat-rich regions; while CHG and CHH methylation are primarily found in transposable elements (31, 32). Southern blot analysis of genomic DNA digested with the differentially methylation-sensitive enzymes MspI and HpaII revealed slightly reduced CG methylation of centromeric 180-bp repeat regions in *icu11-3* (Fig. 2*B*), that was reverted to WT CG methylation in a line where *Ds* had retransposed restoring the WT ICU11 allele (*SI Appendix*, Fig. S5*A*). To further investigate the consequence of the observed changes in DNA methylation, we compared expression of a subset of transposons in early-stage seedlings (*SI Appendix*, Fig. S5 *B*–*D*). We observed some slight up-regulation in *icu11-3* compared to WT and *clf-2*, though to a lesser extent than in DNA methylation mutants *ddm1-1* and *cmt3-7*.

To further examine the effect of *icu11-3* on the cytosine methylome, we performed bisulfite sequencing on seedlings of *icu11-3* mutant and the WT progenitor (*SI Appendix*, Table S2). Consistent with the previous report (25), we found a marginal decrease in CG methylation over genes (*SI Appendix*, Fig. S6*A*) and no substantial changes of CG methylation levels over transposons (*SI Appendix*, Fig. S6*B*). The repetitive nature of the pericentromeric regions complicates their analysis, but we observed a slight reduction in CG methylation at cen180 repeats in *icu11-3* (*SI Appendix*, Table S3). From the combination of bisulfite sequencing and Southern analysis we can conclude that a small proportion of the centromeric repeats lose their CG DNA methylation in *icu11-3*. CHG and CHH (H = A, C or T) methylation over transposon sequences were slightly increased in the *icu11-3* mutant (*SI Appendix*, Fig. S6*B*), but these slight changes in the non-CG contexts are consistent with the variations seen among plant tissues and plants grown in different environmental conditions due to the dynamics of non-CG methylation (33, 34). Overall, therefore these data agree with previous observations that *ICU11* function does not have a large impact on DNA methylation (25). The observed effects may be due to indirect effects of increased transcription at ICU11 targets on the wider *Arabidopsis* epigenome.

### *icu11* Perturbs the Vernalization Response in *Arabidopsis*.

**Fig. 3.**
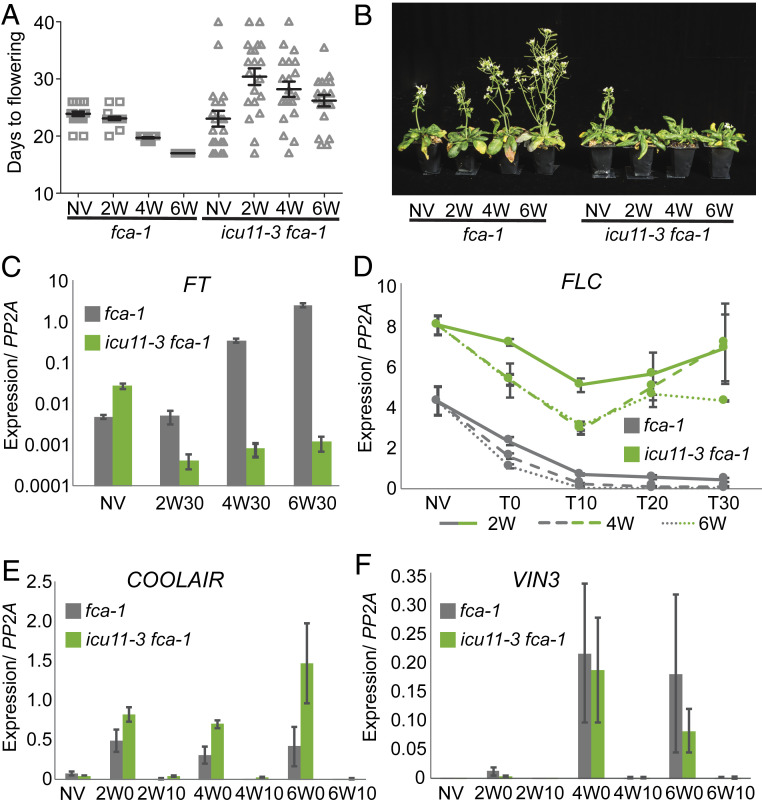
*icu11-3* is defective in vernalization. (*A* and *B*) Flowering is significantly more asynchronous at all time points and significantly delayed in the *icu11-3 fca-1* genotype after 2 wk vernalization (*SI Appendix*, Table S4). Days to flowering counted from the end of a pregrowth period of 14 d in warm conditions (NV), or from the end of pregrowth followed by a 2-, 4-, or 6-wk vernalization treatment. Bars represent mean and SEM (*n* = 20). (*B*) Flowering plants imaged at 25 d postvernalization. (*C*–*F*) Gene expression in the vernalization-requiring *fca1* and *icu11-3 fca-1* backgrounds. Gene expression is normalized to *PP2A*; error bars represent SEM for three biological replicates. (*C*) *FT* expression at 30 d posttreatment is higher in *fca1* than *icu11-3 fca-1*. (*D*) Expression of the flowering repressor *FLC* is higher in *icu11-3 fca1* than *fca-1* before (NV), during (T0), and 10, 20, or 30 d after vernalization (T10–30), reactivating strongly in *icu11-3 fca1* compared with the stable postvernalization repression observed in *fca-1*. Increasing vernalization time (2, 4, and 6 wk) leads to lower and more stably silenced *FLC* expression. (*E*) The antisense RNA *COOLAIR* is expressed at higher levels in *icu11-3 fca1* than *fca-1*. (*F*) Expression of *VIN3*, a cold-induced PRC2 target, is not affected by the *icu11-3* mutation.

To clarify the functional implications of the physical and genetic interaction of ICU11 with PRC2, we asked how the *icu11* mutation affected vernalization, a well understood PRC2-regulated developmental transition. To set a high *FLC* expression state and therefore a vernalization requirement, we crossed *icu11-3* to *fca-1*. *icu11-3 fca-1* plants showed an increased variability in flowering compare to *fca-1* (Fig. 3 *A* and *B* and *SI Appendix*, Table S4). Cold treatment of different durations revealed a clear vernalization defect in *icu11-3 fca-1* plants; flowering was significantly delayed after 2 wk cold treatment (*SI Appendix*, Table S4) and was only marginally accelerated with longer cold. No difference in flowering was observed in the *icu11-3* single mutant following vernalization (*SI Appendix*, Fig. S7).

We collected tissue before vernalization (NV), on the final cold day after 2, 4, or 6 wk cold (T0), and at 10-d intervals (T10, 20, 30) upon return of plants to warm, long-day conditions, allowing us to examine the expression of key regulatory genes in the vernalization-induced flowering pathway. Expression of *FT* in *fca-1* and *icu11-3 fca-1* mirrored the differences observed in flowering time. Without cold, *FT* was expressed at ∼10-fold higher levels in *icu11 fca-1*, even though expression of *FLC* (its repressor) was also high. This presumably reflects misregulation of all Polycomb target genes in an *icu11* background (Fig. 3*C*). Following increasing periods of cold exposure, *FT* expression levels increased in WT plants, but less so in *icu11 fca-1*. In *fca-1*, *FLC* expression decreased quantitatively by prolonged cold and was maintained in the transcriptionally silent state after return to warm conditions. In contrast, in *icu11-3 fca-1, FLC* expression was approximately twofold higher prior to vernalization (Fig. 3*D*), and although expression was reduced by cold, this was not maintained following vernalization. We interpret this as reflecting a reduced ability for the locus to digitally switch to an epigenetically silenced state. Consistent with this, the *icu11-3* mutant showed higher expression of the *FLC* antisense transcript *COOLAIR* during the cold (Fig. 3*E*), but expression of the cold-induced *FLC* repressor *VIN3*, another PRC2 target, was unaffected (Fig. 3*F*).

### ICU11 Functions Directly at *FLC* to Affect the Balance of Histone Modifications.

Vernalization-induced silencing of *FLC* is dependent on PRC2-mediated switching between bistable opposing epigenetic states: an active state, marked by high levels of H3K36me3 at the nucleation region of *FLC*; and a silent state, marked by a peak of H3K27me3 in the nucleation region that accumulates during cold exposure and spreads to cover the entire *FLC* locus upon return to the warm. We had previously shown that H3K4me3 at *FLC* follows transcription rather than functioning as the opposing chromatin state to H3K27me3 (1).

**Fig. 4.**
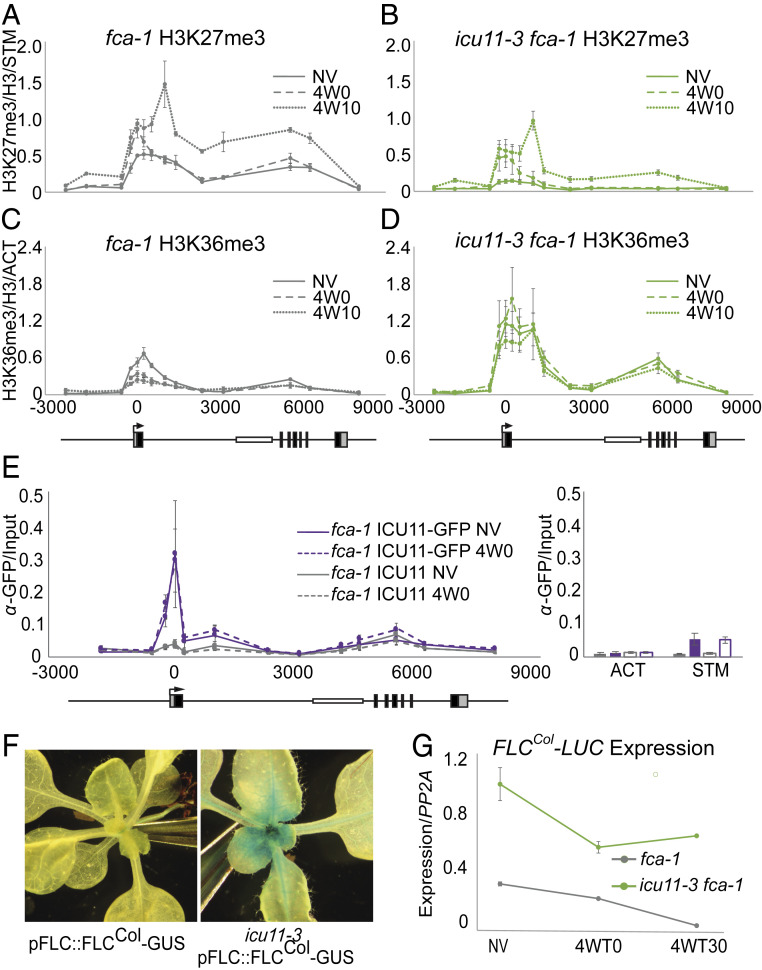
ICU11 influences H3K27me3 and H3K36me3 dynamics at *FLC* through a vernalization timecourse. (*A*–*E*) ChIP analysis of H3K27me3 (*A* and *C*) and H3K36me3 (*B* and *D*) histone modifications and ICU11 localization (*E*) at *FLC*; error bars are SEM of three biological replicates. Abundance, relative to total histone H3 and normalized to *STM* (H3K27me3) or *ACT7* (H3K36me3) was measured in nonvernalized plants, after 4 wk vernalization and vernalization followed by 10 d growth in warm conditions. The *icu11-3 fca-1* mutant (*C* and *D*) has reduced H3K27me3 and increased H3K36me3 relative to the *fca-1* control (*A* and *B*). (*E*) ChIP analysis of ICU11 localization at *FLC* before and after 4 wk vernalization treatment, normalized to input. Purple lines represent plants containing the GFP-tagged ICU11 construct, while gray lines represent control plants with untagged ICU11. Localization of ICU11-GFP at ACT and STM before (solid bar) and after (empty bar) vernalization shown as positive and negative controls. Underlying *A*–*E*, an *FLC* locus schematic showing UTRs (gray boxes), exons (black boxes), and the Ler background MULE insertion in intron one (white box). (*F*) Gus staining of *FLC* expression in FLC^Col-0^-GUS, which lacks the MULE in intron one found in Ler background. Expression of this allele is higher in *icu11-3*, indicating misregulation of the MULE insertion is not responsible for the *FLC* overexpression phenotype. (*G*) Expression of *FLC-LUC* from FLC^Col-0^-LUC (no MULE in intron one) is higher in the *icu11-3* background than WT before and after 4 wk vernalization and shows reactivation after vernalization followed by postcold growth. Error bars are SEM of three biological replicates.

Comparison of *icu11-3 fca-1* compared to *fca-1* over the vernalization time series was used to define the role of ICU11 in *FLC* epigenetic switching. As expected, H3K36me3 was lost from the nucleation region in *fca-1* after 4 wk of cold exposure and replaced by a peak of H3K27me3 that spread to cover *FLC* after 10 d subsequent growth in warm long-day conditions (Fig. 4). In contrast, in *icu11-3 fca-1* the H3K36me3 peak was higher before vernalization and was not lost from the nucleation region following cold exposure or subsequent warm growth conditions. This was coincident with an attenuated accumulation of H3K27me3 accumulation at the locus following cold (Fig. 4). Comparison of H3K27me3 and H3K36me3 levels at *ACT7* and *STM* indicated that histone methylation was relatively stable across time points and between genotypes at control genes (*SI Appendix*, Fig. S8). Thus, ICU11 is required for the cold-induced epigenetic switch between the active H3K36me3 state to the silenced H3K27me3 state at *FLC*.

These analyses had been undertaken using *icu11-3*, a mutation in the Landsberg *erecta* (Ler) background. In L*er*, a Mutator-like transposable element (MULE) in the first intron of *FLC* leads to low expression before vernalization compared with other alleles. We wanted to confirm the effect of ICU11 at *FLC* was on Polycomb switching rather than an influence *FLC* through an effect of the MULE insertion. First, we performed chromatin immunoprecipitation to define sites of association of ICU11 at *FLC*, using a GFP-tagged ICU11 construct transformed into the *icu11-3 fca-1* background. ICU11 was localized at the *FLC* 5′ end coincident with the H3K36me3 enrichment, both before vernalization and at the end of a 4-wk cold treatment (Fig. 4*E*). We also observed a small increase in *ICU11* occupancy over the gene body after 4 wk cold, similar to that seen for other PRC2 components (1). Thus, *FLC* is a direct target of ICU11, colocalizing with PRC2 components before cold and at distinct phases of the cold silencing process. Notably, our ICU11-GFP chromatin immunoprecipitation (ChIP) experiment did not indicate enrichment of ICU11 signal over the MULE.

To confirm our hypothesis that ICU11 does not influence *FLC* via an effect of the MULE, we utilized a *pFLC::FLC-GUS* reporter construct generated using the Col-0 *FLC* allele (*pFLC::FLC*^*Col-0*^*-GUS*), which lacks the MULE. This construct was crossed into the *icu11-3* (Ler) background, and WT and *icu11-3* siblings from an F2 population examined in nonvernalizing conditions. In *icu11-3*, *FLC* expression was strongly up-regulated before cold even in the absence of the MULE (Fig. 4*F*). We used a second Col-0 allele construct, *pFLC::FLC*^*Col-0*^*-LUCIFERASE*, to examine whether the *FLC* reactivation seen following vernalization was also independent of transposon derepression in *icu11-3*. The *pFLC::FLC*^*Col-0*^*-LUCIFERASE* construct was backcrossed into the *icu11-3 fca-1* background. Paralleling our observation of the native *FLC*^*Ler*^ allele in *icu11-3 fca-1* (Fig. 3*D*), we found that *FLC-LUC* expression was higher in the *icu11-3* background than in WT prior to vernalization, was reduced by cold, but that transcription reactivated on return to the warm (Fig. 4*G*). Thus, defective silencing of *FLC* in *icu11-3* is PRC2-dependent and independent of the MULE insertion in *FLC*^*Ler*^.

## Discussion

Here, through analysis of a mutant displaying a range of PcG-impaired phenotypes we identify the ICU11 Fe2+/2 oxoglutarate-dependent oxygenase activity that influences *Arabidopsis* PRC2 regulation. The large 2OGD superfamily in *Arabidopsis*, of which ICU11 is a member, contains prolyl-4-hydroxylases, AlkB DNA repair enzymes and enzymes integral to an array of biosynthetic processes (26). However, *ICU11* and its most closely related sequences form a separate clade within the *Arabidopsis* genome (25), suggesting distinct functionality. Our analysis of global changes and specific defects in the vernalization silencing process suggests that impairment of ICU11 primarily influences active histone marks—especially H3K36me3. A 2-OGD is the catalytically active demethylation domain of Jumonji histone demethylases; however, we could not reproducibly show using in vitro assays that ICU11 has this biochemical activity. Notably, previous studies have similarly struggled to demonstrate recombinant activity in vitro despite clear functional evidence for their function in vivo (35, 36).

The subtle alterations in DNA methylation in the *icu11-3* mutant were unexpected for a mutation predominantly associated with Polycomb silencing. Such pleiotropic changes have not previously been reported for PRC2 mutants in *Arabidopsis*; indeed, a comparable Southern blot analysis indicates that even the severe PRC2 *clf swn* double mutant maintains effective cen180 repeat methylation (37). Thus, we speculate that any DNA methylation *icu11* phenotypes are linked to altered transcriptional activity. ICU11 may work specifically as an H3K36me3 demethylase, influencing directly PRC2 targets and then indirectly a larger set of targets. Alternatively, ICU11 may act as a promiscuous histone demethylase targeting distinct methylation types according to the specificity of its binding partners. However, the robust immunoprecipitation with PRC2 components, but not other chromatin regulators, argues against this. ICU11 may also influence methylation levels of trans factors. For example, the histone demethylase LSD1 demethylates both H3K4 methylation and the DNA methyltransferase Dnmt1 in mouse embryonic stem cells, directly influencing H3K4 and indirectly reducing H3K9me2 (38). Further analysis on defining demethylase activities is clearly important if we are to fully understand epigenetic homeostasis within the *Arabidopsis* genome.

## Materials and Methods

The transposon-tagged lines are in the Landsberg *erecta* genotype. Other material is in the Col-0 genotype. Detailed descriptions of the growth conditions and the experimental procedures used—cloning, qRT-PCR, proteomics, Southern blots, whole-genome methylation analysis, Western blots, chromatin immunoprecipitation, and the in vitro histone demethylation assay—are in *SI Appendix*, *Materials and Methods*.

### Materials and Data Availability.

The full list of mass spectrometry results is provided as Dataset S1. The DNA methylation analysis data have been deposited at Gene Expression Omnibus (accession no. GSE151449). All of the other raw data and materials that support the findings of this study are available from the corresponding authors upon reasonable request.

## Supplementary Material

Supplementary File

Supplementary File
