# Supplementary material for: The Arabidopsis epigenetic regulator ICU11 as an accessory protein of Polycomb Repressive Complex 2
Source: Proc Natl Acad Sci U S A. 2020 Jun 29;117(28):16660–6. doi: 10.1073/pnas.1920621117 (PMC7368280; doi:10.1073/pnas.1920621117)
Supplement: Supplementary File [file pnas.1920621117.sapp.pdf]

PNAS
www.pnas.org

Supplementary Information for

The Arabidopsis epigenetic regulator ICU11 as an accessory protein of Polycomb Repressive Complex 2

Bloomer RH[1*], Hutchison CE[1#], Bäurle I[1@], Walker J[1], Feng X[1], Fang, X[1], Perera P[2], Velanis CN[2], Gümüs S[3], Spanos C[4], Rappsilber J[4,5], Goodrich J[2] and Dean C[1]

[1]Department of Cell & Developmental Biology, John Innes Centre, Colney Lane, Norwich NR47UH, UK

[2]Institute of Molecular Plant Sciences, University of Edinburgh, Daniel Rutherford Building, Max Born Crescent, Edinburgh EH9 3BF, UK

[3]Faculty of Biotechnology, Hochschule Mannheim, Paul-Wittsack-Straße 10, 68163 Mannheim, Germany

[4]Wellcome Centre for Cell Biology, University of Edinburgh, Max Born Crescent, Edinburgh, EH9 3BF, UK

[5]Institute of Biotechnology, Technische Universität Berlin, 13355 Berlin, Germany

Present addresses:

[*]The New Zealand Institute for Plant and Food Research, Gerald Street, Lincoln 7608, New Zealand

[#]William Harvey Research Institute, Charterhouse Square Barts and the London School of Medicine and Dentistry Queen Mary University of London, London EC1M 6BQ, UK

www.pnas.org/cgi/doi/10.1073/pnas.1920621117

[@]Institute for Biochemistry and Biology, University of Potsdam, Am Neuen Palais 10, 14469 Potsdam, Germany

Dame Professor Caroline Dean

Email: caroline.dean@jic.ac.uk

This PDF file includes:

Supplementary text

Figures S1 to S8

Tables S1 to S4

Legends for Dataset S1

SI References

Other supplementary materials for this manuscript include the following:

Dataset S1

Materials and Methods

Plant materials, growth and phenotyping

The *icu11-3* mutant was identified among progeny in a transposon tagging screen using modified maize *Ac*/*Ds* elements, as described previously (1). A stable *icu11-3* mutant was obtained after segregation of the transposase away from the *icu11-3 Ds* insertion. A family of phenotypically-wildtype revertant lines was generated, where the *Ds* insertion in *ICU11* had re-transposed; these revertant lines were selfed to generate stable revertant lines. The progenitor line C12b (2) is used as the wildtype (WT) control line, except where specified as Ler (the original background of the Ac/Ds insertion line; this and all other mutants in this study are in the Ler background, unless otherwise stated). Tagged construct seed lines for proteomic analysis were generous gifts to the Goodrich lab, and have been described elsewhere: pEMF1::EMF1-3XFLAG *emf1-2* (3); 35S:GFP-CLF *clf-50* (4); pSWN::SWN-GFP (5). Seedlings for qPCR, ChIP and proteomics were grown on MS/agar plates without glucose supplementation. Sterilised seeds were sown directly onto plates and stratified for three days at 4°C before being grown in 16h light: 8h dark at 23°C in a walk-in growth room (long day conditions) or 8h light:16h dark at 22°C in a Sanyo growth cabinet (short day conditions). For vernalization qPCR, flowering and ChIP experiments, plants were pre-grown for ten days post-germination under long day conditions then transferred to a walk-in growth room under 8h light:16h dark at 4°C. Following vernalization on plates, seedlings were pricked out onto soil in 24-cell trays and grown on in long day conditions to score flowering time or to harvest tissue for late (T20, T30) qPCR timepoints. Flowering time was scored as the number of days in warm short- or long-day conditions, following pre-growth ; and cold treatment, to first visible buds. The difference in mean time to flowering following vernalization was assessed with Welch's ANOVA and Dunnett's correction for multiple testing using Graphpad (Prism); while the synchronicity of flowering following vernalization was assessed by comparing coefficients of variation for each sample using Feltz and Miller's asymptotic test (6), implemented in the R package cvequality, and a Bonferroni correction for multiple testing.

Cloning *ICU11*

Genomic DNA fragments adjacent to each end of the *Ds* insertion were identified by inverse PCR (iPCR), and subsequent cloning and sequencing of iPCR products. This approach revealed *Ds* terminal sequences, and an 8bp duplication of the Arabidopsis insertion site sequence, which is characteristic of *Ds* insertions. A BLAST search with the genomic DNA fragment at the 5' end of the *Ds* element identified BAC clone F19G10 (Genbank accession number AF000657). Comparison of sequences from

iPCR with cosmid libraries, 3' and 5' RACE and cDNA cloning were used to predict and confirm the presence of an open reading frame (ORF) of nine exons, encoding a 397-amino acid protein of unknown function (At1G22950).

Generation of *ICU11* constructs

We synthesized the *ICU11* locus in three components for GoldenGate cloning: a 2071bp promoter fragment; the 1875kb genomic *ICU11* coding region, and 2168kb of downstream terminator sequence. Native BsaI sites within the coding sequence were 'domesticated' to simplify GoldenGate digestion and ligation without altering the encoded amino acid sequence. Two versions of the coding sequence were designed: the first with a 3' overhang for scarless ligation to the terminator fragment; and the second with a 3' overhang complementary to the ENSA GoldenGate C-terminal tag construct set. Fragments were assembled into a Gateway-compatible vector backbone by simultaneous digestion/ligation reactions with BsaI-HF (New England Biolabs) and T4 DNA ligase (New England Biolabs) using the following cycling conditions: 25 cycles of 37°C for three minutes then 16°C for four minutes; following cycling, the samples were heated to 50°C for five minutes and 80°C for a further five minutes to denature all enzymes. Following assembly, plasmids were cloned into *E. coli* DH5-a and screened positive assembly colonies identified by PCR and sequencing. Correctly assembled constructs were recombined by Gateway LR cloning (Invitrogen) into the binary vector backbone pSLJ-dest, a Gateway-compatible vector based on pSLJ755I6 (7) and transformed into *Agrobacterium tumifaciens* C58 by triparental mating. The *icu11-3* mutant was transformed with native ICU11 and two tagged ICU11 lines, ICU11-GFP and ICU11-HA; positive transformants were identified by BASTA selection of T1 plants grown on soil. Positive transformants showed complementation of morphological phenotypes in the T1 generation, and pooled T2 seed was used to confirm complementation by quantitative RT-PCR of ICU11 target genes.

qRT-PCR

RNA extractions were performed using the hot phenol method (8), followed by removal of contaminating DNA with TURBO DNase (Invitrogen) according to the manufacturer's protocol. RNA was precipitated with sodium acetate and ethanol; after quantification, 2µg of RNA was used in a 10uL cDNA synthesis reaction with Superscript III and either oligo d(T) or gene-specific primers (Table S1). The resulting cDNA was diluted 10-fold for qPCR with SybrGreen2, and results analysed using the ΔΔc(T) method (three biological replicates, each with three technical replicates per data point).

Proteomics

Inflorescence tissue (1-3g) was harvested from plants grown in continuous light at 16°C. Tissue was frozen in liquid nitrogen, finely ground using pestle and mortar, and resuspended in two volumes of IP buffer (10mM Tris pH 7.5, 150 mM NaCL, 0.5% Igepal, 1% Triton) containing 1X protease inhibitor cocktail (Roche) and 0.1mM PMSF or 1mM Pefabloc (Roche). The subsequent IP was performed as described previously (9, 10) with the exception that FLAG-tagged samples were immunoprecipitated using anti-FLAG M2 gel affinity beads (Sigma), and HA-tagged samples with μMACS HA tagged protein isolation kit (Miltenyi Biotech). Proteins were separated by NuPAGE Novex 4-12% Bis-Tris gel, (Life Technologies, UK), in NuPAGE buffer (MES) (Life Technologies, UK) and visualised using InstantBlueTM stain (Sigma Aldrich, UK). The stained gel bands were excised and de-stained with 50mM ammonium bicarbonate (Sigma Aldrich, UK) and 100% (v/v) acetonitrile (Sigma Aldrich, UK) and proteins were digested with trypsin, as previously described (11). Briefly, proteins were reduced in 10 mM dithiothreitol (Sigma Aldrich, UK) for 30 min at 37°C and alkylated in 55 mM iodoacetamide (Sigma Aldrich, UK) for 20 min at ambient temperature in the dark. They were then digested overnight at 37°C with 12.5 ng µL-1 trypsin (Pierce, UK).

Following digestion, samples were diluted with equal volume of 0.1% TFA and spun onto StageTips as described by Rappsilber *et al* (12). Peptides were eluted in 40 µL of 80% acetonitrile in 0.1% TFA and concentrated down to 1 µL by vacuum centrifugation (Concentrator 5301, Eppendorf, UK). Samples were then prepared for LC-MS/MS analysis by diluting them to 5 µL with 0.1% TFA. LC-MS-analyses were performed on a Q Exactive Mass Spectrometer (Thermo Fisher Scientific, UK) coupled on-line, to an Ultimate 3000 RSLCnano Systems (Dionex, Thermo Fisher Scientific, UK). Peptides were separated on a 50 cm EASY-Spray column (Thermo Fisher Scientific, UK) assembled in an EASY-Spray source (Thermo Fisher Scientific, UK) and operated at a constant temperature of 50oC. Mobile phase A consisted of 0.1% formic acid (Sigma Aldrich, UK) in deionised water while mobile phase B consisted of 80% acetonitrile and 0.1% formic acid. Peptides were loaded onto the column at a flow rate of 0.3 µL min-1 and eluted at a flow rate of 0.2 µL min-1 according to the following gradient: 2 to 40% mobile phase B in 90 min (150 min for ICU11-HA and ICU11-GFP samples), then to 95% in 11 min and returned at 2% 6 min after.

FTMS spectra were recorded at 70,000 resolution (scan range 350-1400 m/z) and the ten most peaks with charge ≥ 2 of the MS scan were selected with an isolation window of 2.0 Thomson for MS2 (filling 1.0E6 ions for MS scan, 5.0E4 ions for MS2, maximum fill time 60 ms, dynamic exclusion for 60 s). Only

ions with charge between 2 and 6 were selected for MS2. The normalized collision energy for the HCD fragmentation (13) that was used was set at 27.

The MaxQuant software platform (14) version 1.5.2.8 was used to process raw files and search was conducted against the complete *Arabidopsis thaliana* Database (Uniprot, released October 2014), using the Andromeda search engine (15). The first search peptide tolerance was set to 20 ppm while the main search peptide tolerance was set to 4.5 pm. Isotope mass tolerance was 2 ppm and maximum charge to 7. Maximum of two missed cleavages were allowed. Carbamidomethylation of cysteine was set as fixed modification. Oxidation of methionine and acetylation of the N-terminal as well as the Gly-Gly (diglycil) on lysine were set as variable modifications. For peptide and protein identifications FDR was set to 1%.

Southern blots and whole-genome DNA methylation analysis

DNA was extracted from Ler, progenitor lines C12b (containing the *Ds* element in an streptomycin resistance fusion) and 1962-1 (containing a stabilized *Ac* element that contained a deletion in the 5' untranslated region for increased activity), stable *icu11-3* mutant and revertant lines using a modified CTAB method (16); 0.5-2µg per sample was digested using five units of enzyme (*Hpa*II or *Msp*I, NEB) per microgram of DNA in either Cuts-all or manufacturer's buffer at the manufacturer's recommended temperature. Following digestion, a phenol:chloroform cleanup was used to remove enzymes. Southern blotting used DNA run on an agarose gel overnight at 1.5V/cm, denatured, and transferred to a nylon membrane (Amersham Hybond-N or -N^+). DNA was crosslinked to the membrane by irradiation (Stratalinker, Stratagene) and subsequently probed with a cen180 (17) plasmid, radiolabelled with 32P following the method of Feinberg and Vogelstein (18).

For genome-wide methylation analysis, single-end bisulfite-sequencing libraries for Illumina sequencing were constructed with Ovation Ultralow Methyl-Seq Library Systems (Nugen, 0336) and EpiTect Fast Bisulfite Conversion (Qiagen, 59802) kits according to the kit protocols, except for the incorporation of two rounds of bisulfite conversion. Sequencing was performed on site at the John Innes Centre using a Nextseq 500. DNA methylation analysis was performed as previously described (19) with the TAIR10 genome.

Western blots, histone and ICU11 chromatin immunoprecipitation

Nuclei for western blots were extracted using one of two methods: for H3K27me3 and H3K36me3, histones were extracted from 2g of 14-day old WT and icu11-3 seedlings using Honda buffer, lysis buffer, sonication and benzonase treatment as described previously (20). For H3K4me1-3, histones were extracted using an acid hydrolysis method (21); briefly, finely ground seedlings were resuspended in ice cold NIB buffer (0.25M sucrose, 60mM KCl, 15mM NaCl, 5mM MgCl2, 1mM CaCl2, 15mM PIPES pH6.8, 0.8% Triton X-100, 0.1mM Pefabloc (Roche), protease inhibitor cocktail). Slurry was filtered twice through a single layer of Miracloth, then pelleted by centrifugation at 10,000g for 20 minutes at 4oC. Basic proteins were extracted twice from the pellet by resuspension in 0.4M sulfuric acid followed by centrifugation at 14,000rpm. Pooled supernatants were then precipitated in 20x vol acetone overnight at 4oC, pelleted and dried before resuspension in 4M urea. Following extraction, serial two-fold dilutions were run on 4-12% Bis-Tris gels (Invitrogen), transferred to nitrocellulose membranes and probed overnight with primary antibodies against H3 (Abcam ab1791), H3K4me1 (Abcam ab8895), H3K4me2 (Abcam ab7766), H3K4me3 (Abcam ab8850), H3K36me3 (Abcam ab9050) and H3K27me3 (Millipore 07-449) and anti-mouse or anti-rabbit antibodies as appropriate (AmershamTM ECLTM Anti-Mouse IgG (GE, NA931), AmershamTM ECLTM Anti-Rabbit IgG (GE, NA934)). Histone ChIP experiments were performed as described previously (20), using anti-H3 (Abcam ab1791), anti-H3K36me6 (Abcam ab9050) and anti-H3K27me3 (Millipore 07-449) and protein A Dynabeads (Invitrogen).

To quantify the western blot signal for H3K36me3, the original raw image was converted into a JPEG file format and the picture mode was changed to “Grayscale” using Photoshop. The image was then loaded into ImageJ. The “rectangle” tool from ImageJ was used to draw a frame around each band and the grey mean value was measured. The same frame was used to measure the grey mean value for all the bands as well as for the background. The value of background was then subtracted from that of each band. The resulting value of H3K36me3 was then normalized to that of H3.

The pICU11::ICU11-GFP construct in icu11-3 was crossed to fca-1 background to establish a vernalization requirement, and *fca-1* and *fca-1 pICU11::ICU11-GFP* grown for ChIP experiments to confirm localization of ICU11 at *FLC* before and during vernalization. ChIP was undertaken as described previously (22); briefly, extracted nuclei were resuspended in RIPA buffer and sonicated (Agilent Bioruptor) to give fragments of ~500bp. The chromatin extract was cleared by high-speed sonication, and extracts were incubated at 4°C with anti-GFP antibody (Abcam ab290) and Protein A agarose/salmon sperm DNA beads (Millipore 16-157) for immunoprecipitation. Following clean up and DNA recovery, qPCR was performed with primers across the *FLC* locus (Table S1). STM and ACT were used to compare protein enrichment at positive and negative controls, respectively.

In vitro histone demethylation assay

ICU11 cDNA sequence was amplified using primers ICU-expr1F and ICU-expr1R (Table S1) and cloned into the pGEX-4T-1 expression vector using XhoI and SmaI restriction sites, generating a GST-tagged ICU11 protein expression construct. pGEX::ICU11-GST was transformed into the *E. coli* BL21 strain for protein expression and induced by treatment of cultures with IPTG; ICU11-GST was purified on Glutathione Sepharose 4B beads (Sigma), eluted with glutathione elution buffer (50 mM Tris-HCl, 10 mM reduced glutathione, pH 8.0) and used fresh for histone demethylation assays with either Arabidopsis histone extract (purified in Honda buffer, sonicated and and resuspended in monococcal nuclease buffer (200mM Tris pH8, 50mM NaCl, 2.5mM $CaCl_2$, 5mM $MgCl_2$) and benzonase-treated as above for western blot), or with calf thymus histones (Sigma). Four serial two-fold dilutions of the ICU11-GST purification were incubated with an equal amount of either native Arabidopsis or calf thymus histones for 1h at 37°C in histone demethylase buffer (50mM HEPES pH8, 1mM α-ketoglutarate, 2mM ascorbate, 14mM $Fe(NH_4)_2(SO_4)_2$). Following incubation, reactions were stopped by addition of Laemmli/SDS buffer followed by a 10 minute boiling treatment at 95°C; the reactions were run on 4-12% Bis-Tris gels (Invitrogen), transferred to nitrocellulose membranes and probed overnight with primary antibodies against H3 (Abcam ab1791) and H3K36me3 (Abcam ab9050) as for western blots above.

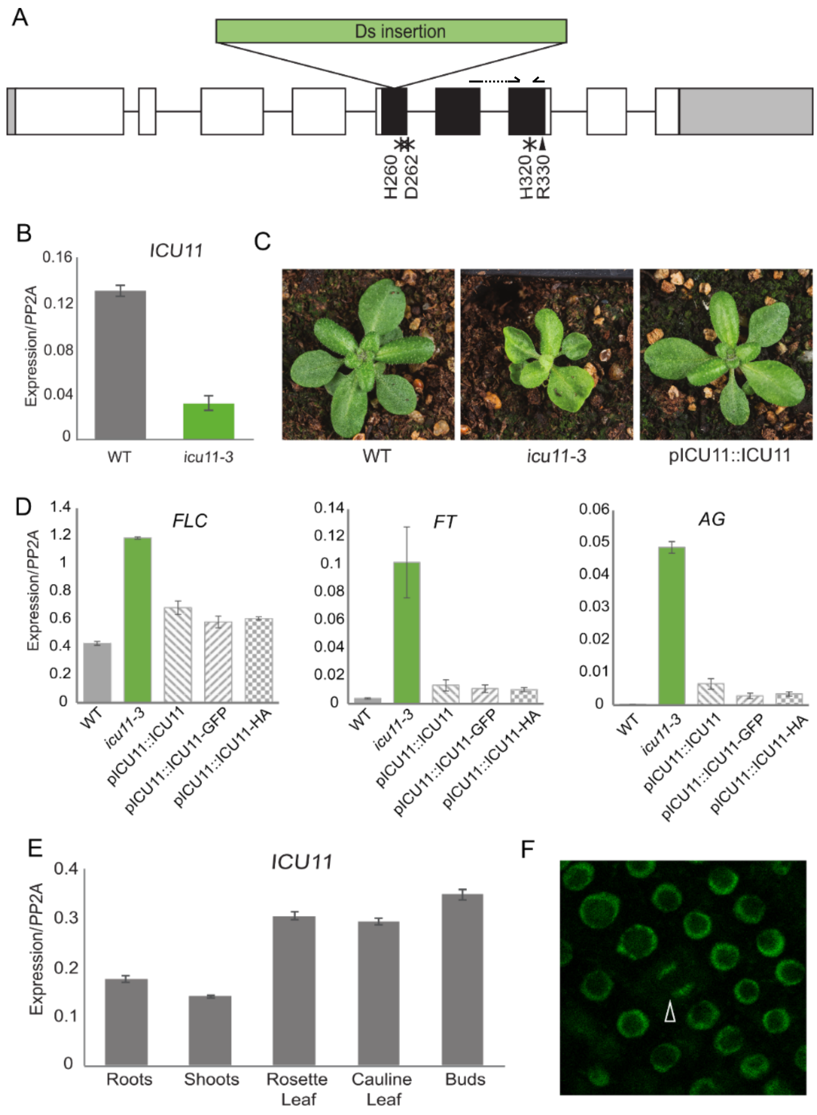

Figure S1: Characterisation of the *icu11-3* mutation. (A) Schematic representation of the *ICU11* locus with 5' and 3' UTRs as grey bars, exonic regions as white bars, and the predicted Fe2+/2-oxoglutarate-dependent oxygenase (2OG) domain indicated in black with conserved residue positions noted with *(Fe2+ binding residues) and Δ (2-oxoglutarate binding). The position of the DS transposon insertion in *icu11-3*, which generates a premature stop codon, is indicated by the green bar. The forward primer used for *ICU11* expression analysis spans the exon 6/7 boundary (black forward arrow, with splice site indicated by dashed line) while the reverse primer falls in exon 7 (black reverse arrow). (B) Expression of *ICU11* is reduced in the *icu11-3* mutant. Expression of *ICU11* is normalised to the housekeeping gene *PP2A*. (C) Complementation of *icu11-3* with a single copy of *ICU11* under its own promoter is sufficient to recover wildtype seedling morphology. (D) Complementation of *icu11-3* with a single copy of *ICU11*, with or without C-terminal GFP or HA tag, reduces overexpression of PRC2 target genes to near-wildtype levels. Expression of PRC2 targets *FT, FLC* and *AG* is normalised to the housekeeping gene *PP2A* (E) *ICU11* is broadly expressed across tissues (separated roots and shoots at 14 days post-germination; rosette and cauline leaves; inflorescence buds) during wildtype Arabidopsis development. (B,D,E) Bars represent the mean of three biological replicates and error bars the standard error of the mean. (F) ICU11-GFP expression in emerging roots shows the protein is primarily localised to nuclei, and remains associated with the DNA during mitosis (white triangle).

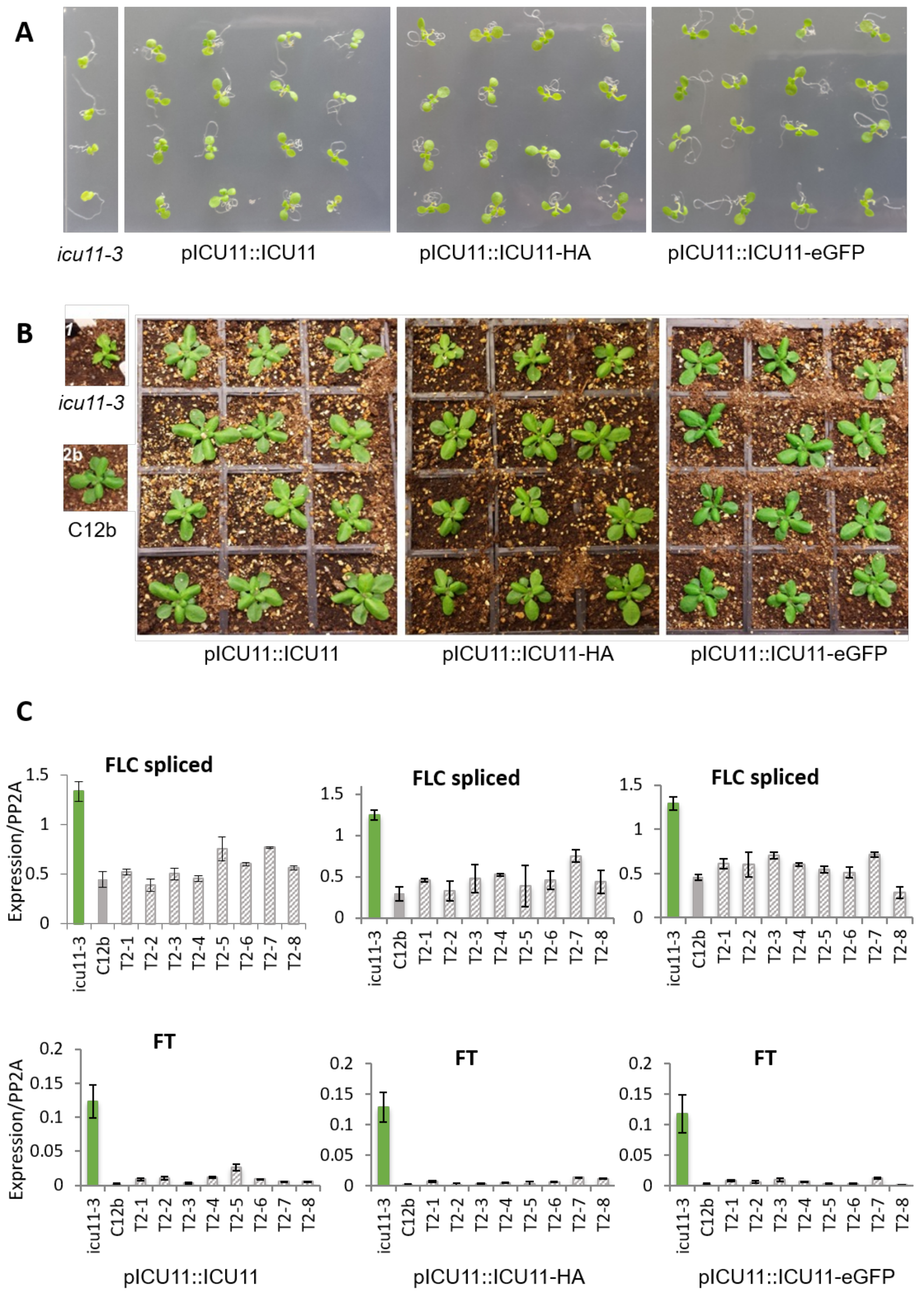

Figure S2: Characterisation of T2 *icu11-3* mutant rescue with untagged and C-terminal HA- and eGFP-tagged ICU11 proteins. (A) Complementation of *icu11-3* with either pICU11::ICU11 untagged, C-terminal HA-tagged or eGFP-tagged constructs rescues the curled cotyledon phenotype in independent T2 lines (B) Complementation of *icu11-3* with either pICU11::ICU11 untagged, C-terminal HA-tagged or eGFP-tagged constructs rescues the rosette size and leaf furling phenotypes of the mutant (C) Independent, single-copy insertion of either pICU11::ICU11 untagged, C-terminal HA-tagged or eGFP-tagged constructs is sufficient to restore *FLC* and *FT* expression in two-week old T2 seedlings to that of the parental line C12b compared with *icu11-3.* Expression is normalized to he housekeeping gene PP2A; bars represent the mean of three biological replicates and error bars the standard error of the mean.

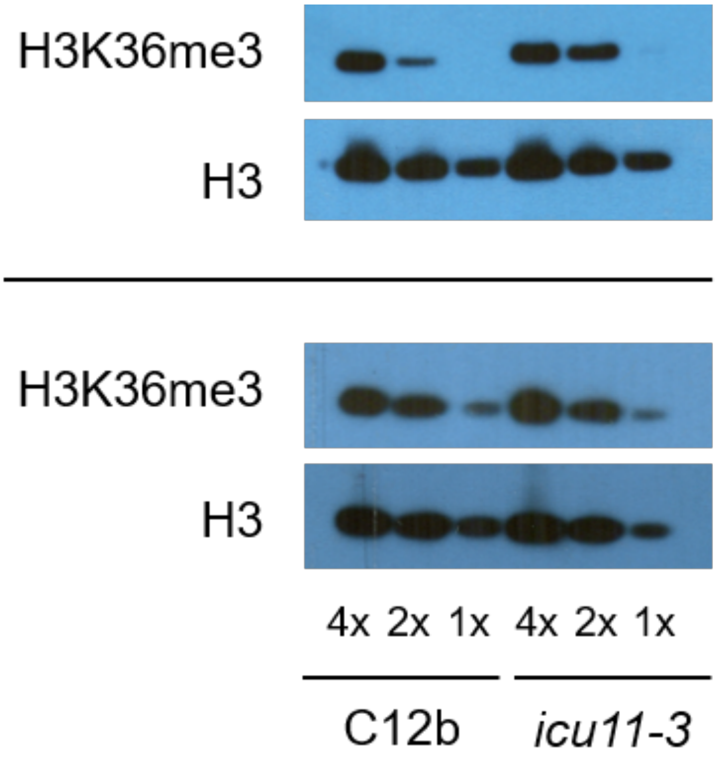

Figure S3: *icu11-3* reproducibly shows elevated H3K36me3 in comparison to wildtype. Two additional replicate western blots to those pictured in Figure 2, with independently grown two-week old seedlings, reproducibly demonstrate that H3K36me3 is elevated in *icu11-3* compared with wildtype C12b seedlings. Three two-fold serial dilutions were loaded for each replicate and sample, with the same dilutions run in parallel to probe for H3 as a loading control.

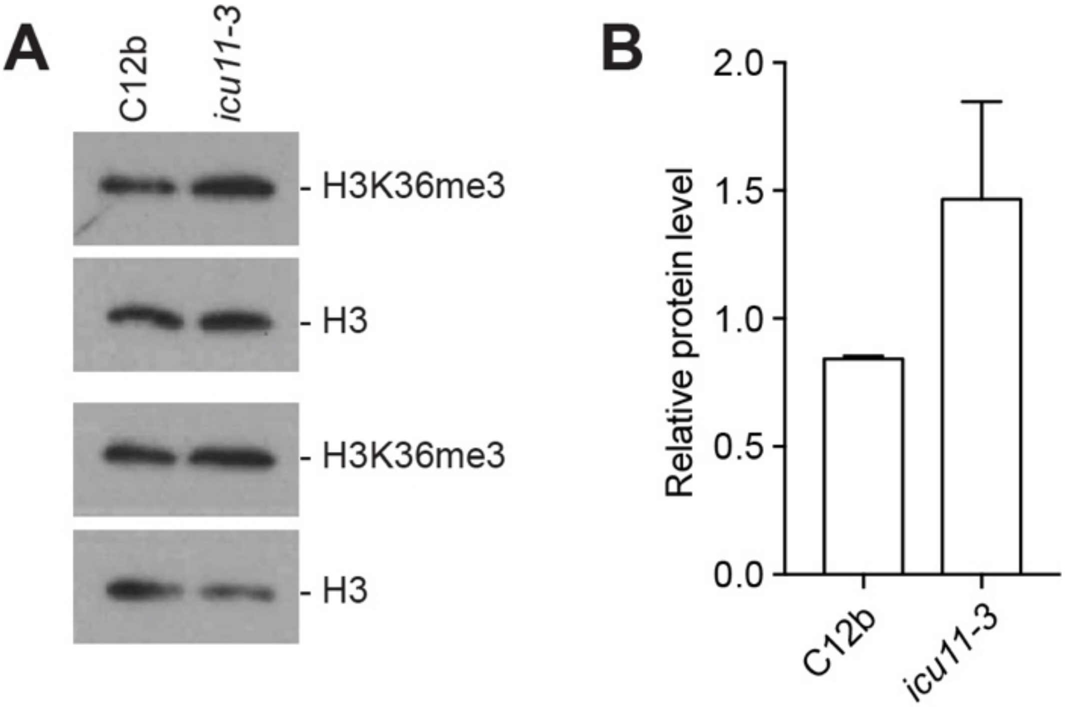

Figure S4: ***The icu11-3*** **mutant shows about 1.5-fold increase in global H3K36me3 level.** (A) The level of global H3K36me3 in the indicated genotypes as determined by western blot. (B) The intensity of H3K36me3 relative to H3 in the indicated genotypes. Note that the data are from the two biological replicates shown in (A). Data are presented as the mean ± SEM.

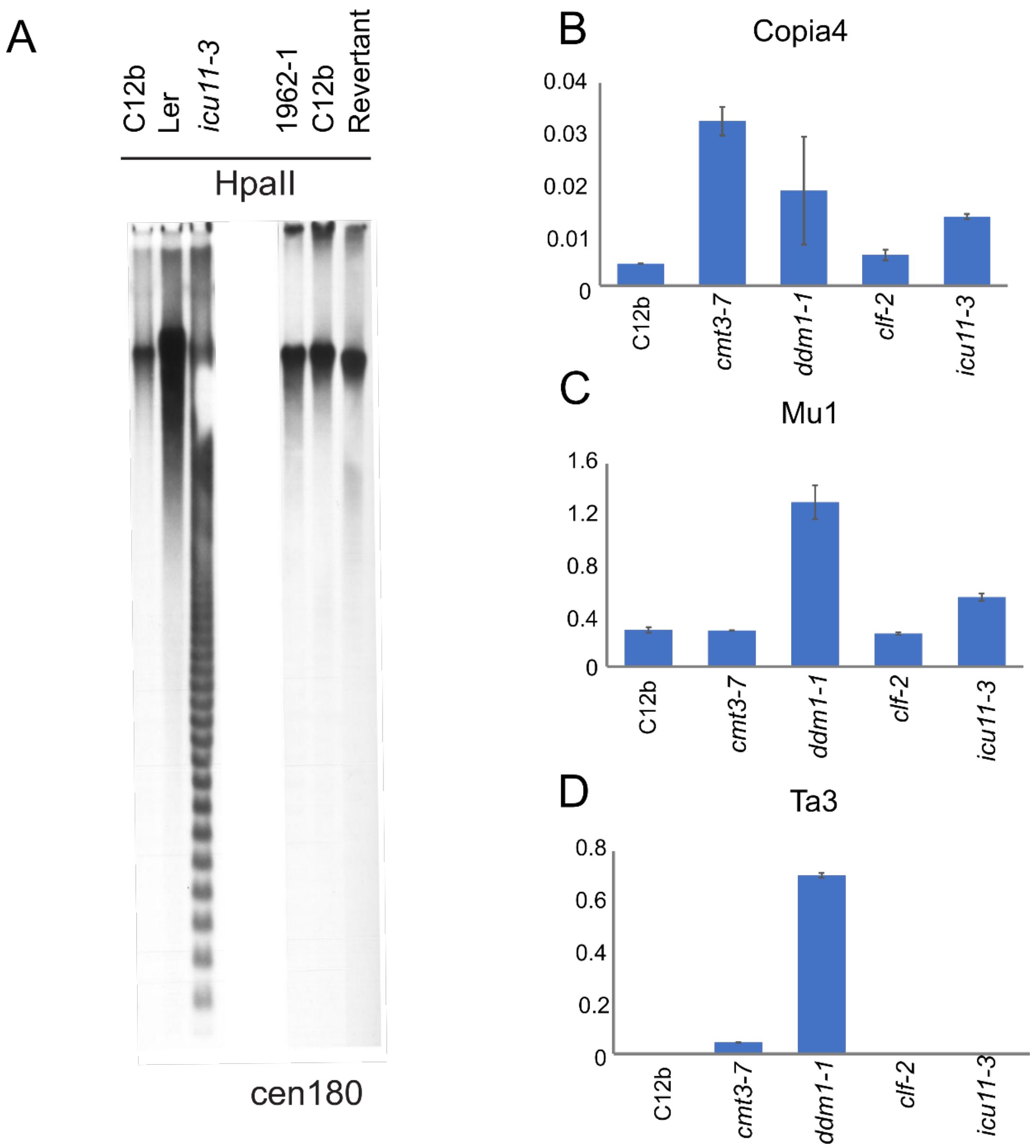

Figure S5: The *icu11-3* mutation exhibits restricted DNA hypomethylation and elevated expression of transposable elements. (A) Probing with a cen180 centromeric repeat probe reveals "laddering" after HpaI digestion, indicative of DNA hypomethylation, only with *icu11-3* mutants and not with parental Ac/Ds lines C12b and 1962-1, the C12b progenitor line Ler, or in revertant lines that have lost the Ds transposon and *icu11-3* morphological phenotype. (B-D) Expression of selected transposable elements is slightly elevated in 14 day post-germination *icu11-3* seedlings relative to wild-type or *clf-2* PRC2 mutants, but much less so than for DNA methylation mutants *cmt3-7* and -*ddm1-1.* Bars show the mean of three biological replicates and error bars standard error of the mean.

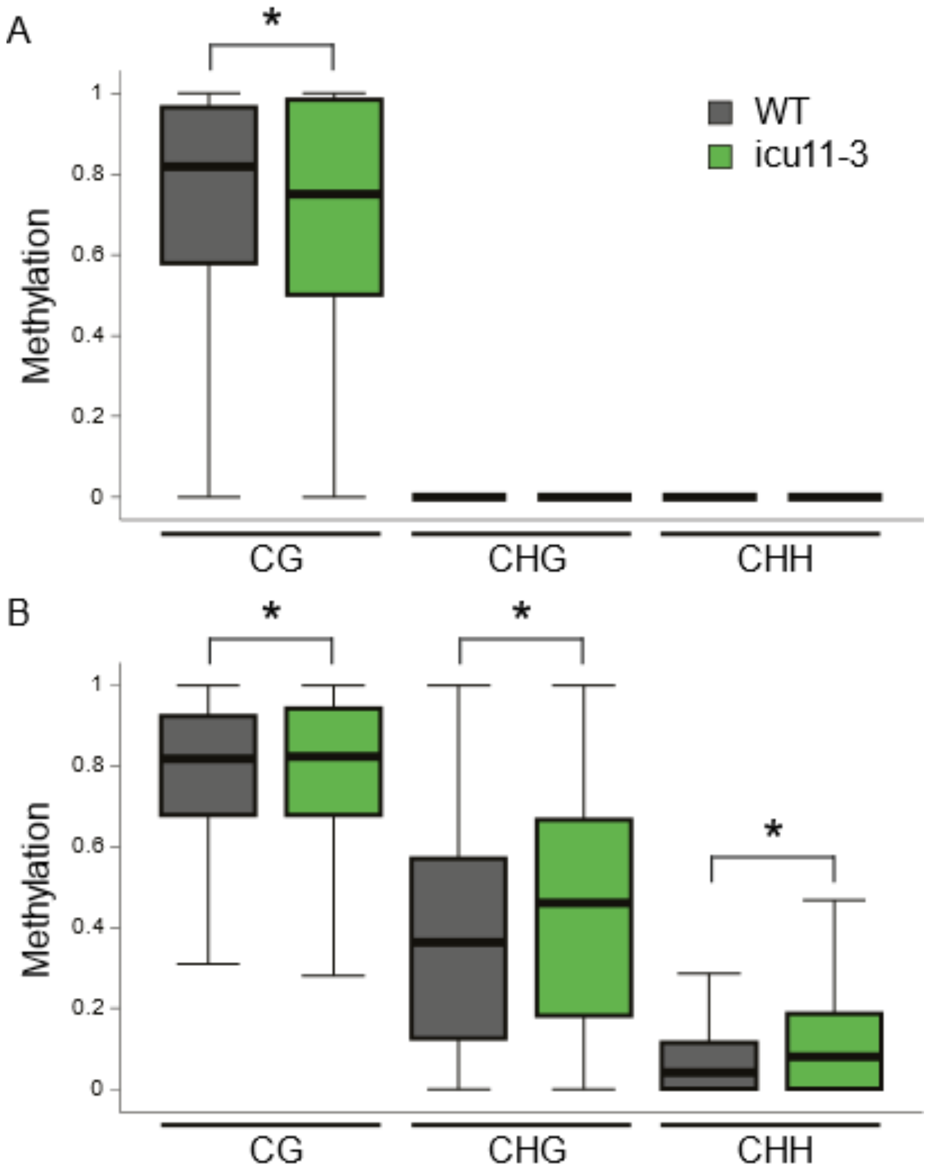

Figure S6: The *icu11-3* mutant exhibits limited variation in DNA methylation across the genome. Box plots depict CG, CHG and CHH methylation for 50 bp windows with a cytosine sequence coverage of at least 10 and at least 50% CG methylation in either wildtype (grey plots) or *icu11-3* mutants (green plots) overlapping either TAIR10-annotated genes (28,775 genes, A) or transposons (31,189 transposons, B). Each box encloses the middle 50% of the distribution, with the horizontal line marking the median and vertical lines marking the minimum and maximum values falling within 1.5 times the height of the box. * p<0.0001, Kolmogorov–Smirnov test.

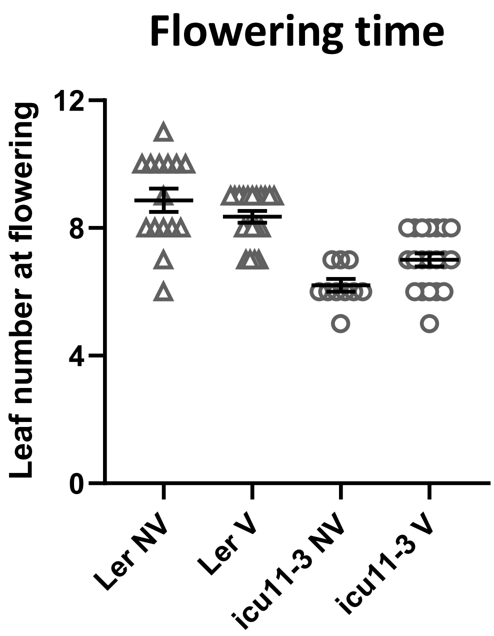

Figure S7: The *icu11-3* single mutant shows no acceleration of flowering in response to vernalization. Ler (open triangles) exhibits slightly earlier flowering following a four week seed vernalization treatment (V) compared with no vernalization (NV); while in contrast flowering of the the *icu11-3* single mutant (open circles) is not accelerated by vernalization (V) compared with no vernalization (NV). Error bars indicate mean and standard error of the mean (n=10-20 individual plants).

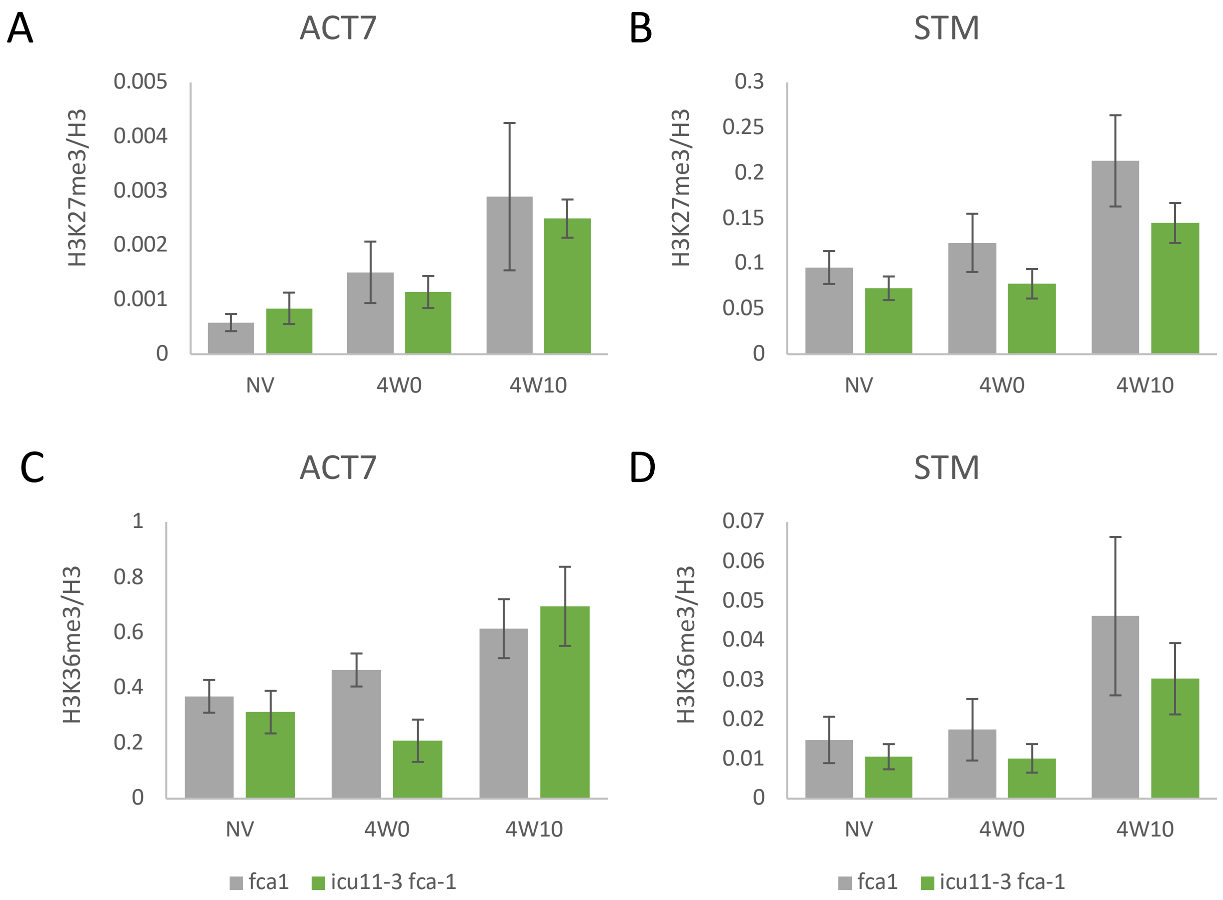

Figure S8: Histone methylation at control genes in wildtype and mutant genotypes and across time points. H3K27me3 (A, B) and H3K36me3 (C, D) levels normalised to total H3 in mutant and wild type. Bars are the mean of three biological replicates and error bars are +/- SEM.

Table S1: Primers used in this work

Experiment	Primer	Sequence 5'-3'	Notes
ICU11 expression cloning	ICU11_EcoExpr_F	ATCGTTCCCGGGTGAAATGTGCAATC	
	ICU11_EcoExpr_R	ATTGCTCTCGAGTGTTTACTCGGCACATG	
qPCR	PP2A-F	ACTGCATCTAAAGACAGAGTTCC	
	PP2A-R	CCAAGCATGGCCGTATCATGT	
	ICU11-F	GATAGCACTGAAAAGGAAGTTTATG	
	ICU11-R	CACAAAATCAAGTTGGCTCGG	
	FT-F	CTGGAACAACCTTTGGCAAT	
	FT-R	AGCCACTCTCCCTCTGACAA	
	FLC-spliced-F	AGCCAAGAAGACCGAACTCA	
	FLC-spliced-R*	TTTGTCCAGCAGGTGACATC	
	FLC-unspliced-F	CGCAATTTTCATAGCCCTTG	
	FLC-unspliced-R	CTTTGTAATCAAAGGTGGAGAGC	
	AG-F	CCGATCCAAGAAGAATGAGCTCTT	Primer source (23)
	AG-R	CATTTTCAGCTATCTTTGCACGAA	Primer source (23)
	LFY-F	CCCACCAAGGTGACGAACCA	Primer source (23)
	LFY-R	ACAGTGAACGTAGTGTCGCATT	Primer source (23)
	AP1-F	CGACGTCAATACAAACTGGTCGAT	Primer source (23)
	AP1-R	CTTTAGGGCTCATTGCTTGCA	Primer source (23)
	AP3-F	CCCTAACACCACAACGAAGGAGAT	Primer source (23)
	AP3-R	GTTTCCTCTTGGTTTCTTGCATTC	Primer source (23)
	STM-F	GAAGCTTACTGTGAAATGCTCG	Primer source (24)
	STM-R	AACCACTGTACTTGCGCAAGAG	Primer source (24)
	CP2_F	CAGCAACAAAGGCAGATGAAAC	
	CP2-R	TGCACCATAGAAGCATATTGACC	
	COPIA4-F	GCAAACGCTGCATCTGAAGTT	Primer source (25)
	COPIA4-R	TGTTGCGAACGAAATGGTAGTC	Primer source (25)

	MU1-F	TTGAATGAGGAACCACATACTTG	Primer source (25)
	MU1-R	GAATTAATGACTTCGCTCTTGCT	Primer source (25)
	Ta3-F	GATTCTTACTGTAAAGAACATGGCATTGAGAGA	Primer source (26)
	Ta3-R	TCCAAATTTCCTGAGGTGCTTGTAACC	Primer source (26)
	VIN3-F	TGCTTGTGGATCGTCTTGTCA	
	VIN3-R	TTCTCCAGCATCCGAGCAAG	Primer source (27)
	COOLAIR-F	TGCATCGAGATCTTGAGTGTATGT	
	COOLAIR-R	ACGTCCCTGTTGCAAAATAAGC	
	FLC-qPCR-Fwd-LUC-F	GATTACGTCGCCAGTCAAGTAAC	
ChIP	FLC_-2429_F	ATCCAGAAAAGGGCAAGGAG	Histone and ICU11 ChIP
	FLC_-2376_R	CGAATCGATTGGGTGAATG	Histone and ICU11 ChIP
	FLC_-1708_F	TGGAGGGAACAACCTAATGC	Histone and ICU11 ChIP
	FLC_-1639_R	TCATTGGACCAAACCAAACC	Histone and ICU11 ChIP
	FLC_-501_F	ACTATGTAGGCACGACTTTGGTAAC	Histone and ICU11 ChIP
	FLC_-381_R	TGCAGAAAGAACCTCCACTCTAC	Histone and ICU11 ChIP
	FLC_-158_F	GCCCGACGAAGAAAAAGTAG	Histone and ICU11 ChIP
	FLC_-56_R	TCCTCAGGTTTGGGTTCAAG	Histone and ICU11 ChIP
	FLC_48_F	CGACAAGTCACCTTCTCCAAA	Histone and ICU11 ChIP
	FLC_205_R	AGGGGGAACAAATGAAAACC	Histone and ICU11 ChIP
	FLC_307_F	GGCGGATCTCTTGTTGTTTC	Histone and ICU11 ChIP
	FLC_393_R	CTTCTTCACGACATTGTTCTTCC	Histone and ICU11 ChIP
	FLC_543_F	CGTGCTCGATGTTGTTGAGT	Histone and ICU11 ChIP
	FLC_700_R	TCCCGTAAGTGCATTGCATA	Histone and ICU11 ChIP
	FLC_1035_F	CCTTTTGCTGTACATAAACTGGTC	Histone and ICU11 ChIP
	FLC_1148_R	CCAAACTTCTTGATCCTTTTTACC	Histone and ICU11 ChIP
	FLC_1424_F	TTGACAATCCACAACCTCAATC	Histone and ICU11 ChIP
	FLC_1561_R	TCAATTTCCTAGAGGCACCAA	Histone and ICU11 ChIP
	FLC_1884_F	AGCCTTTTAGAACGTGGAACC	Histone and ICU11 ChIP
	FLC_2062_R	TCTTCCATAGAAGGAAGCGACT	Histone and ICU11 ChIP
	FLC_2356_F	AGTTTGGCTTCCTCATACTTATGG	Histone and ICU11 ChIP

FLC_2451_R	CAATGAACCTTGAGGACAAGG	Histone and ICU11 ChIP
FLC_3088_F	GGGGCTGCGTTTACATTTTA	Histone and ICU11 ChIP
FLC_3224_R	GTGATAGCGCTGGCTTTGAT	Histone and ICU11 ChIP
FLC_MUT5_F	CTTGACAAGTTGAAGGAGTCTG	ICU11 ChIP only
FLC_3478_R	AGTAGCACATCTGAATTTCCAC	ICU11 ChIP only
FLC_3889_F	CTTTTTCATGGGCAGGATCA	Histone and ICU11 ChIP
FLC_4069_R	TGACATTTGATCCCACAAGC	Histone and ICU11 ChIP
FLC_4213_F	AGAACAACCGTGCTGCTTTT	Histone and ICU11 ChIP
FLC_4360_R	TGTGTGCAAGCTCGTTAAGC	Histone and ICU11 ChIP
FLC_5030_F	CCGGTTGTTGGACATAACTAGG	Histone and ICU11 ChIP
FLC_5135_R	CCAAACCCAGACTTAACCAGAC	Histone and ICU11 ChIP
FLC_5534_F	TGGTTGTTATTTGGTGGTGTG	Histone and ICU11 ChIP
FLC_5649_R	ATCTCCATCTCAGCTTCTGCTC	Histone and ICU11 ChIP
FLC_5948 F	CGTGTGAGAATTGCATCGAG	Histone and ICU11 ChIP
FLC_6066 R	AAAAACGCGCAGAGAGAGAG	Histone and ICU11 ChIP
FLC_6768_F	TTGTAAAGTCCGATGGAGACG	Histone and ICU11 ChIP
FLC_6838_R	ACTCGGCGAGAAAGTTTGTG	Histone and ICU11 ChIP
STM exon F	GCCCATCATGACATCACATC	Histone and ICU11 ChIP
STM exon R	GGGAACTACTTTGTTGGTGGTG	Histone and ICU11 ChIP
ACTIN 16_F	GATATTCAGCCACTTGTCTGTG	Histone and ICU11 ChIP
ACTIN 100_R	CTTACACATGTACAACAAAGAAGG	Histone and ICU11 ChIP

Table S2: Mean genomic coverage and DNA methylation for wild-type and mutant Ler seedlings mapped to TAIR10

	Nuclear				Chloroplast
Sample	Coverage	CG, %	CHG, %	CHH, %	Overall CHH, %
C12b	5.8	16.4	4.2	1.4	0.2
icu11-3	6.5	17.7	5.9	2.5	0.2

Chloroplast CHH methylation is an estimate of cytosine nonconversion rate and other errors. Mean methylation is calculated by averaging methylation of individual cytosines in each context.

Table S3: A comparison of methylation between *icu11-3* and c12b at centromeric repeat sequences. Significant difference was determined for each cytosine (p<0.05, fisher exact test).

Centromeric repeat sequence	Number of CG sites	Number of CG sites with significantly higher methylation in *icu11-3*	Number of CG sites with significantly higher methylation in c12b	Centromeric repeat sequence with cytosines showing significant difference between *icu11-3* and c12b	Median CG methylation difference across the repeat (*icu11-3*-c12b)
178	6	**2**	**4**	Yes	-0.013
285	4	0	**4**	Yes	-0.011
286	8	**2**	**2**	Yes	0.029
287	4	0	**2**	Yes	-0.060
288	10	0	0	No	x
289	4	0	**3**	Yes	-0.068
290	6	0	**5**	Yes	-0.063
291	6	**4**	**1**	Yes	0.027
292	6	**1**	**5**	Yes	-0.018
706	4	0	**2**	Yes	-0.008

Centromeric repeat sequence	Number of CHG sites	Number of CHG sites with significantly higher methylation in *icu11-3*	Number of CHG sites with significantly higher methylation in WT	Centromeric repeat sequence with cytosines showing significant difference between *icu11-3* and WT	Median CHG methylation difference across the repeat (mutant-WT)
178	3	**3**	0	Yes	0.059
285	2	**2**	0	Yes	0.026
286	3	**1**	**1**	Yes	0.002
287	1	**1**	0	Yes	0.016
288	3	0	**1**	Yes	-0.150
289	6	**3**	**2**	Yes	0.051
290	2	**1**	0	Yes	-0.003
291	3	**2**	0	Yes	0.037
292	6	**2**	**2**	Yes	0.007
706	3	**3**	0	Yes	0.170

Centromeric repeat sequence	Number of CHH sites	Number of CHH sites with significantly higher methylation in *icu11-3*	Number of CHH sites with significantly higher methylation in WT	Centromeric repeat sequence with cytosines showing significant difference between *icu11-3* and WT	Median CHH methylation difference across the repeat (mutant-WT)
178	47	**29**	**1**	Yes	0.018
285	50	**38**	**1**	Yes	0.020

286	53	**13**	**6**	Yes	0.012
287	56	**28**	**2**	Yes	0.032
288	54	**20**	**2**	Yes	0.004
289	55	**41**	**3**	Yes	0.038
290	53	**22**	**10**	Yes	0.006
291	51	**39**	**1**	Yes	0.057
292	55	**44**	0	Yes	0.023
706	17	**16**	0	Yes	0.106

Table S4: Significance of differences in flowering time and synchronicity following vernalization. The difference in means was tested with Welch's ANOVA and Dunnett's correction for multiple comparisons; while the difference in synchronicity was tested by comparing coefficients of variation with Feltz and Miller's asymptotic test and a Bonferroni correction for multiple testing.

	Mean flowering time		Coefficient of variation	
Comparison	Adjusted p-value	Significant	p-value	Significant
fca1 NV vs 2WV	0.6781	N	1	N
fca1 NV vs 4WV	<0.0001	Y	1	N
fca1 NV vs 6WV	<0.0001	Y	1	N
icu11-3 fca1 NV vs 2WV	0.0074	Y	1	N
icu11-3 fca1 NV vs 4WV	0.1042	N	1	N
icu11-3 fca1 NV vs 6WV	0.3141	N	1	N
fca1 NV vs *icu11-3 fca1* NV	0.9995	N	2.04×10^{-6}	Y
fca1 2WV vs *icu11-3 fca1* 2WV	0.0007	Y	5.48×10^{-7}	Y
fca1 4WV vs *icu11-3 fca1* 4WV	<0.0001	Y	7.15×10^{-12}	Y
fca1 6WV vs *icu11-3 fca1* 6WV	<0.0001	Y	1.42×10^{-13}	Y

Dataset S1: Full IP-MS results for Table 1, showing all proteins identified in replicated ICU11-GFP, ICU11-3xHA and reciprocal pulldowns with EMF1, CLF and SWN.

SI References

1. I. Bancroft, J. D. G. Jones, C. Dean, Heterologous transposon tagging of the *DRL1* locus in Arabidopsis. *Plant Cell* **5**, 631–638 (1993).

2. I. Bancroft, C. Dean, Transposition Pattern of the Maize Element *Ds* in *Arabidopsis thaliana*. *Genetics* **134**, 1221–1229 (1993).

3. M. Calonje, R. Sanchez, L. Chen, Z. R. Sung, *EMBRYONIC FLOWER1* Participates in Polycomb Group-Mediated *AG* Gene Silencing in Arabidopsis. *Plant Cell* **20**, 277–291 (2008).

4. D. Schubert, *et al.*, Silencing by plant Polycomb-group genes requires dispersed trimethylation of histone H3 at lysine 27. *EMBO J.* **25**, 4638–4649 (2006).

5. M. de Lucas, *et al.*, Transcriptional Regulation of Arabidopsis Polycomb Repressive Complex 2 Coordinates Cell-Type Proliferation and Differentiation. *Plant Cell* **28**, 2616 (2016).

6. C. J. Feltz, G. E. Miller, An asymptotic tests for the equality of coefficients of variation from k populations. *Stat. Med.* **15**, 647–658 (1996).

7. J. D. G. Jones, *et al.*, Effective vectors for transformation, expression of heterologous genes, and assaying transposon excision in transgenic plants. *Transgenic Res* **1**, 285–297 (1992).

8. M. S. Box, V. Coustham, C. Dean, J. S. Mylne, Protocol: A simple phenol-based method for 96-well extraction of high quality RNA from Arabidopsis. *Plant Methods* **7**, 7 (2011).

9. M. Derkacheva, *et al.*, Arabidopsis MSI1 connects LHP1 to PRC2 complexes. *EMBO J.* **32**, 2073–2085 (2013).

10. S. C. Liang, *et al.*, Kicking against the PRCs - A Domesticated Transposase Antagonises Silencing Mediated by Polycomb Group Proteins and Is an Accessory Component of Polycomb Repressive Complex 2. *PLoS Genet.* **11**, e1005660–e1005660 (2015).

11. A. Shevchenko, M. Wilm, O. Vorm, M. Mann, Mass Spectrometric Sequencing of Proteins from Silver-Stained Polyacrylamide Gels. *Anal. Chem.* **68**, 850–858 (1996).

12. J. Rappsilber, Y. Ishihama, M. Mann, Stop and Go Extraction Tips for Matrix-Assisted Laser Desorption/Ionization, Nanoelectrospray, and LC/MS Sample Pretreatment in Proteomics. *Anal. Chem.* **75**, 663–670 (2003).

13. J. V. Olsen, *et al.*, Higher-energy C-trap dissociation for peptide modification analysis. *Nat. Methods* **4**, 709 (2007).

14. J. Cox, M. Mann, MaxQuant enables high peptide identification rates, individualized p.p.b.-range mass accuracies and proteome-wide protein quantification. *Nat. Biotechnol.* **26**, 1367 (2008).

15. J. Cox, *et al.*, Andromeda: A Peptide Search Engine Integrated into the MaxQuant Environment. *J. Proteome Res.* **10**, 1794–1805 (2011).

16. C. Dean, C. Sjodin, T. Page, J. Jones, C. Lister, Behaviour of the maize transposable element *Ac* in *Arabidopsis thaliana*. *Plant J.* **2**, 69–81 (1992).

17. J. M. Martinez-Zapater, M. A. Estelle, C. A. Somerville, A highly repeated DNA sequence in *Arabidopsis thaliana*. *Mol. Gen. Genet. MGG* **204**, 417–423 (1986).

18. A. P. Feinberg, B. Vogelstein, A technique for radiolabelling DNA restriction endonuclease fragments to high specific activity. *Anal.Bichem.* **132**, 6–13 (1991).

19. C. A. Ibarra, *et al.*, Active DNA demethylation in plant companion cells reinforces transposon methylation in gametes. *Science* **337**, 1360–1364 (2012).

20. H. Yang, M. Howard, C. Dean, Antagonistic roles for H3K36me3 and H3K27me3 in the cold-induced epigenetic switch at Arabidopsis *FLC*. *Curr. Biol. CB* **24**, 1793–7 (2014).

21. J. P. Jackson, *et al.*, Dimethylation of histone H3 lysine 9 is a critical mark for DNA methylation and gene silencing in Arabidopsis thaliana. *Chromosoma* **112**, 308–315 (2004).

22. H. Yang, *et al.*, Distinct phases of Polycomb silencing to hold epigenetic memory of cold in Arabidopsis. *Science* **357**, 1142-1145 (2017).

23. J. M. Barrero, R. González-Bayón, J. C. del Pozo, M. R. Ponce, J. L. Micol, *INCURVATA2* encodes the catalytic subunit of DNA Polymerase alpha and interacts with genes involved in chromatin-mediated cellular memory in *Arabidopsis thaliana*. *Plant Cell* **19**, 2822–2838 (2007).

24. S. V. Spinelli, A. P. Martin, I. L. Viola, D. H. Gonzalez, J. F. Palatnik, A Mechanistic Link between STM and CUC1 during Arabidopsis Development. *Plant Physiol.* **156**, 1894 (2011).

25. T. Tsuchiya, T. Eulgem, An alternative polyadenylation mechanism coopted to the Arabidopsis *RPP7* gene through intronic retrotransposon domestication. *Proc. Natl. Acad. Sci.* **110**, E3535 (2013).

26. C. Kammel, *et al.*, Arabidopsis DEAD-Box RNA Helicase UAP56 Interacts with Both RNA and DNA as well as with mRNA Export Factors. *PLOS ONE* **8**, e60644 (2013).

27. J. I. Qüesta, J. Song, N. Geraldo, H. An, C. Dean, Arabidopsis transcriptional repressor VAL1 triggers Polycomb silencing at *FLC* during vernalization. *Science* **353**, 485–488 (2016).
